# A Chronic Pain Self-Management Mobile App (Dolodoc): Cross-Sectional Acceptability Study

**DOI:** 10.2196/77163

**Published:** 2026-05-14

**Authors:** Julie Guebey, Laetitia Gosetto, Benno Rehberg, Christian Lovis, Frederic Ehrler, Aude Molinard-Chenu

**Affiliations:** 1 Division of Medical Information Science University Hospital of Geneva Geneva Switzerland; 2 Anesthesiology University Hospital of Geneva Geneva Switzerland; 3 Medecine Faculty University of Geneva Geneva Switzerland; 4 Division of Consultative Psychiatry and Crisis Intervention University Hospital of Geneva Geneva Switzerland

**Keywords:** self-management, chronic pain, user experience, digital health, mobile health, mHealth

## Abstract

**Background:**

Approximately 19% of adults in Europe are affected by chronic pain, which reduces the quality of life. Pain-management mobile health (mHealth) apps offer a promising solution for self-management, but user engagement and adherence can limit their clinical impact. User experience design and research play a crucial role in optimizing usability and long-term adoption of digital health interventions.

**Objective:**

This study aims to evaluate the user experience of Dolodoc, a mobile app for chronic pain self-management, using a mixed methods approach that assesses acceptability through a content quality survey and examines use by analyzing overall use patterns.

**Methods:**

A cross-sectional acceptability study of the main content of Dolodoc was conducted among patients with chronic pain recruited from the Geneva University Hospitals pain center and through snowball sampling. Participants rated 84 evidence-based self-management strategies by using a 5-point Likert scale based on 5 acceptability criteria: understandability, motivation, feasibility, relevance, and alignment with the related quality-of-life dimension. To reduce participant fatigue and avoid random responses, the 84 strategies were randomly divided across survey versions. Use was assessed through metrics collected over 6 months with Piwik PRO Analytics to observe real-world use behaviors among Dolodoc users.

**Results:**

In the acceptability study, 33 participants rated the self-management strategies positively across all dimensions. On a scale from –2 to 2, the strategies were well understood (mean 1.47, SD 0.76), motivational (mean 1.12, SD 0.96), feasible (mean 1.01, SD 1.05), relevant (mean 0.99, SD 1.09), and aligned with the dimensions (mean 1.33, SD 0.89). The use study demonstrated that 60% (486/802) of the patients used Dolodoc only once, indicating that long-term adherence remains a challenge. Within Dolodoc, pain tracking, useful links, and medication logging were the most actively used features.

**Conclusions:**

This study highlights the gap between acceptability and long-term adherence to mHealth solutions. Improving personalization and accessibility could increase user engagement and long-term adherence. Future iterations of the app should incorporate tailored interventions and real-time feedback mechanisms. In addition, leveraging a digital navigation follow-up could facilitate user adoption and sustained engagement.

## Introduction

Chronic pain is a major health care problem that affects about 19% of adults in Europe [1]. This pernicious symptom affects patients worldwide and across many dimensions of their quality of life [2], underscoring the need to develop new approaches to care. Chronic pain often forces individuals to explore multiple approaches to alleviate pain [3]. Managing chronic pain involves an integrative medicine approach based on pharmaceutical or nonpharmaceutical treatments, psychological support, and self-management strategies [4]. Optimizing self-management is paramount in the promotion of self-efficacy [5], which stands out as a powerful lever [6] to improve clinical outcomes related to chronic pain, such as disability, affective distress, and pain severity [7].

In this context, digital mobile health (mHealth) solutions represent a promising sector [8]. There is a growing number of smartphone apps that leverage these innovations to improve the care trajectories of patients with pain [9,10]. In fact, mobile apps for chronic pain management have demonstrated benefits, including improvements in quality of life and pain severity [11,12]. However, we postulate that their effectiveness depends on how well they address the needs and perceptions of the target users. Motivational factors are key to patient engagement in self-management [13] and multimodal treatment [14]. However, long-term engagement and adherence have emerged as key limiting factors for digital interventions in medicine [15,16]. One way to increase adherence and engagement of target users is user experience (UX) design, which aims to clarify the understanding of the human-product interaction, identifying key components and dimensions to improve design practices [17]. It serves as an essential framework at every stage of the design process, aiming to enhance the overall quality of the final product based on the needs of the target user [18]. UX design in health innovations aligns with the contemporary concept of person-centered medicine, which prioritizes patient engagement and individualized care [19,20]. Commonly used methods in UX studies include use metrics analysis, user interviews, usability testing, surveys, and behavioral analytics. These methods can be applied at various stages of the innovation process, ensuring adaptability to different research and design needs. Mixed methods studies facilitate understanding of UX, enabling better adoption and increased effectiveness of digital mHealth tools [21]. Globally, UX studies aim to understand 2 main characteristics of an mHealth solution: acceptability and usability [22].

Dolodoc is a mobile app designed to facilitate self-management of chronic pain. It features pain self-management strategies focusing on 7 quality-of-life dimensions, the ability to add information about the user’s pain, and the ability to share a summary of pain-related activities and outcomes with health care professionals [23]. A recent study shows significant gaps between the functionality being developed and end-user expectations, highlighting the need for close collaboration among designers, developers, and users throughout the co-design process [24].

In this study, we describe users’ perceptions of acceptability and study their perceptions of the information contained in Dolodoc using 5 selected acceptability criteria based on previous literature: understandability [25], motivation [26], feasibility [26], relevance [27], and alignment with the related quality-of-life dimension. Acceptability is a multifaceted construct reflecting the extent to which individuals delivering or receiving a health intervention consider it appropriate based on their anticipated or experienced cognitive and emotional responses [28]. Usability can be inferred from use studies that examine how systems are used in natural contexts. The aim is to observe and analyze how users interact with a product, service, or technology in their everyday or professional environments, paying particular attention to behaviors, routines, frequency of use, and changes in practice over time. It is mostly used in studies to understand the adoption and effective use of technologies, beyond mere usability performance [29]. In this study, we observe the use habits of real-world users. Overall, this work aims to describe key UX outcomes of an mHealth solution for chronic pain using a mixed methods approach.

## Methods

### Study Design

This mixed methods cross-sectional study assesses UX outcomes of a chronic pain self-management mobile app, including acceptability through content quality assessments by users and use through global use metrics.

### Ethical Considerations

This study was submitted to the Geneva Cantonal Commission for Research Ethics and was deemed to fall outside the scope of the Swiss Federal Act on Research involving Human Beings (Human Research Act); therefore, formal ethics committee approval was not required. All participants were informed about the purpose of the study, and informed written consent was obtained prior to participation. Participation was voluntary, and participants could withdraw at any time without consequence. All data were collected and pseudonymized and handled in accordance with applicable data protection regulations. No personally identifiable information was collected, and confidentiality of participants was strictly maintained. No financial compensation was provided to participants for their participation in this study.

### Mobile App Description

Dolodoc is a mobile app developed to assist patients in self-managing chronic pain. It provides self-management strategies and encourages users to maintain a digital journal to dynamically monitor their pain and related quality-of-life indicators. The development and implementation of this digital tool have been described elsewhere [30], and they follow UX design principles for optimization. The core content of the app consists of a library of 84 evidence-based self-management strategies that users can test in real life, tracking their effects within the app on various pain severity indicators, including quality-of-life measures. More details about the app, including illustrative examples, are available in Multimedia Appendix 1 and in previous publications [23,30]. The strategies were developed through consultations with a focus group of patients with chronic pain and professionals specializing in chronic pain care. Participants first discussed the impact of pain on their daily life in various contexts. From these discussions, 7 quality-of-life dimensions emerged: mood, social support, sleep, work, intimacy, relaxation, and daily activities. Drawing on these dimensions and scientific evidence, a team of psychologists developed motivational pain-management strategies: each quality-of-life dimension corresponds to 4 to 20 strategies, totaling 84 self-management strategies. The self-management strategies were reviewed at the time by a pain specialist and a communication expert.

### Participants

#### Acceptability Study

Participants in the content evaluation survey were eligible for inclusion if they were aged 18 years or older, had chronic pain, were fluent in French, and were able to provide informed consent. Participants were primarily recruited from the pain center at the University Hospital of Geneva, and additional patients meeting the inclusion criteria were recruited through snowball sampling.

There was no requirement to download or use the app to participate, as the study focuses on its content rather than the app itself. It allowed the study to include feedback from experienced users, novices, and nonusers alike. To ensure that each participant fully understood the framework within which the strategies were provided, a standardized contextual presentation of the mobile app was given before participants shared their opinions.

#### Use Study

As detailed in the following section, the use study included data from anonymous Dolodoc users. These data reflect the behavior of all Dolodoc users throughout the study period, not just the participants who took part in the evaluation interviews.

### Setting, Recruitment, and Data Collection

#### Acceptability Study: Content Quality Assessment by Users

Recruitment took place from June to September 2024. Some participants were recruited passively through flyers in the waiting room, but most were recruited actively by members of the pain center team. All 84 strategies were randomly divided into 5 groups to reduce the total duration of the survey. On the basis of the time of their recruitment, participants were allocated to a random sample of 5 to 22 strategies. This methodological choice was made to reduce participants’ burden. In this study, we evaluated the content of the app, not the app itself. Participants were asked to complete an online questionnaire covering demographics, pain characteristics (duration and impact on the 7 quality-of-life dimensions included in Dolodoc), and an evaluation of each sampled strategy. Each strategy was assessed by 5 to 10 participants against 5 acceptability criteria: understandability, motivational impact, feasibility, relevance, and alignment with the relevant quality-of-life dimension (refer to Multimedia Appendix 2 for an example). Responses were collected on a 5-point Likert scale ranging from “strongly disagree” to “strongly agree,” allowing for a quantitative understanding of participants’ perceptions of each strategy.

#### Use Study: Real-World Use Metrics

Use data were obtained from the Piwik PRO Analytics Suite (Piwik PRO GmbH), an online usage analytics platform that collects Dolodoc use metrics via anonymous tracking using cookies and session data. Data were collected over 6 months, from January to June 2024.

A cookie (named _pk_id) is collected to determine if a user is recurring and to calculate the number of visits and the duration of each session. Another cookie (named _pk_ses) is used to indicate whether a session is active. The information contained in these cookies is sent to Piwik with each event during the use of Dolodoc (eg, a page view).

The unique identifier from the _pk_id cookies is used as a proxy to identify unique visitors. At the same time, session data are extracted from _pk_ses, including operating system, session number, page title, time spent on the page, and session duration. Although most of our analyses included both iOS and Android users, the number of sessions per visitor could not be calculated for iOS users, likely because on iOS, _pk_id is deleted after 30 minutes of inactivity due to Apple’s stricter cookie management. As a result, Piwik always identifies iOS users as unique visitors, preventing us from distinguishing between recurring and unique visitors.

### Statistical Methods

Descriptive and inferential statistics were applied depending on the nature of the data. Microsoft Excel, Jupyter Notebook [31], and Orange Data Mining (University of Ljubljana) [32] were used for data analysis and visualization.

## Results

### Acceptability Study

#### Demographics and Pain-Related Characteristics

**Figure 1:**
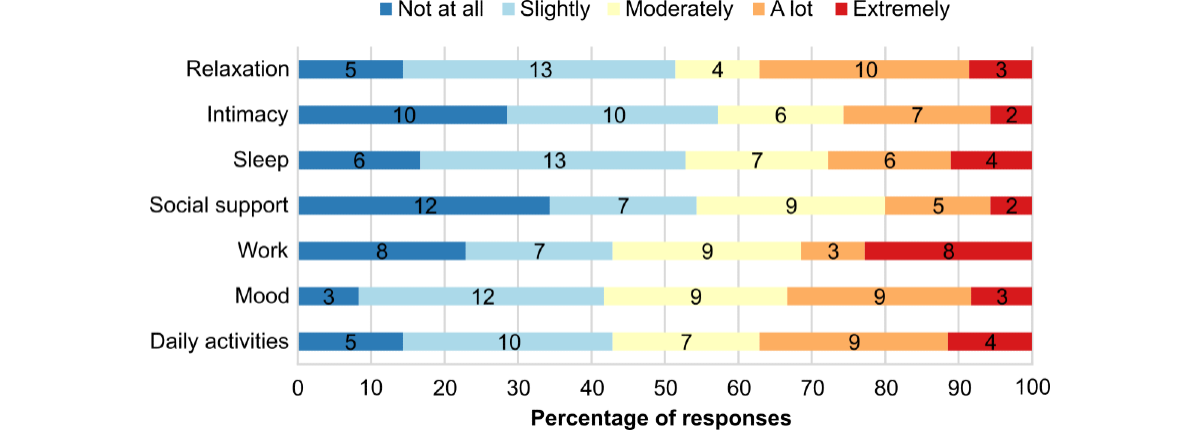
Impact of pain on the 7 quality-of-life dimensions targeted in the self-management strategies incorporated in the Dolodoc mobile app, as reported by the 33 participants. The number of assessments in each subfraction (35-36 in total) is indicated within the bars; 5 missing values were excluded.

A total of 33 participants (n=20, 60.6% female and n=13, 39.4% male individuals) were enrolled in the study. A total of 3 (9.1%) participants evaluated 2 different subsamples of strategies; therefore, the total number of assessments per question in the survey is up to 36. For these 3 (9.1%) recurrent participants, we minimized intraindividual variability using only their most recent responses for demographic variables. The participants’ ages ranged from 23 to 79 years, with a mean age of 38.1 (SD 15.63) years. Most participants reported chronic pain for more than 1 year (n=24, 72.7%), while 9 (27.3%) participants reported chronic pain for less than 1 year. Each quality-of-life dimension was affected to varying degrees, but overall, quality-of-life dimensions were not homogeneously affected by chronic pain in our sample (*χ*^2^_24_=21.4; *P*<.001; Figure 1). These results support the idea of an individual profile in the impact of pain on quality of life.

#### Content Evaluation Survey

**Figure 2:**
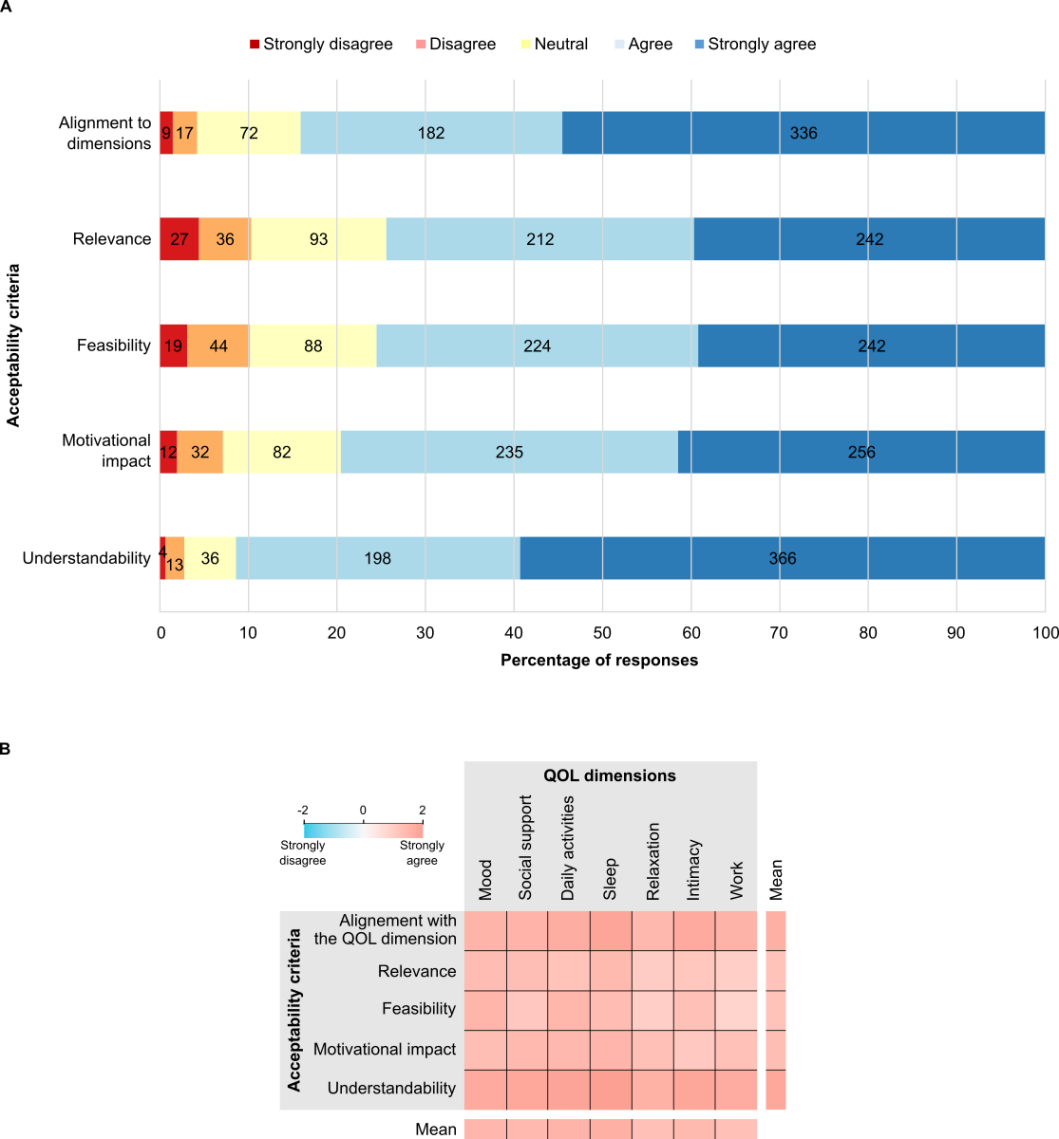
Pain-management strategies in Dolodoc are well perceived by users across 5 acceptability criteria and 7 quality-of-life (QOL) dimensions. (A) Bar plot showing the distribution of ratings across 5 acceptability criteria. The number of assessments in each subfraction is indicated within the bars (B) Heat map displaying the average rating score for acceptability criteria and QOL dimensions (n=3077 ratings in total).

Overall, the content of Dolodoc was well perceived by participants: on a Likert scale from –2 to 2 (from strongly disagree to strongly agree), the average rating across all acceptability criteria was 1.18. Moreover, of the 84 strategies, 62 (73.8%) received strong agreement (mean >1), whereas 22 (26.2%) were agreed upon (mean >0), demonstrating that acceptability criteria were globally fulfilled according to our user sample. More specifically, understandability was the highest-rated acceptability criterion (mean 1.47) while relevance (mean 0.99) and feasibility (mean 1.01) received the lowest ratings. The strategies were also perceived as motivational (mean 1.12) and aligned with the specific dimensions (mean 1.33; Figure 2A). Each acceptability criterion received between 610 and 617 ratings. In this study, missing values accounted for 0.26% (8/3085) of the data because the online questionnaire did not require respondents to answer all questions before submitting the survey.

To precisely assess perceptions of the strategies in the context of well-being, we calculated a mean rating for each of the 7 previously described quality-of-life dimensions, based on each acceptability criterion (Figure 2B). The mean scores range between 1 (agree) and 2 (strongly agree), with values from 1.08 for the “relaxation” dimension and 1.40 for the “sleep” dimension. The detailed, numbered results are provided in Multimedia Appendix 3. These results suggest a positive consensus among participants regarding Dolodoc self-management strategies within all quality-of-life dimensions.

### Use Study

**Figure 3:**
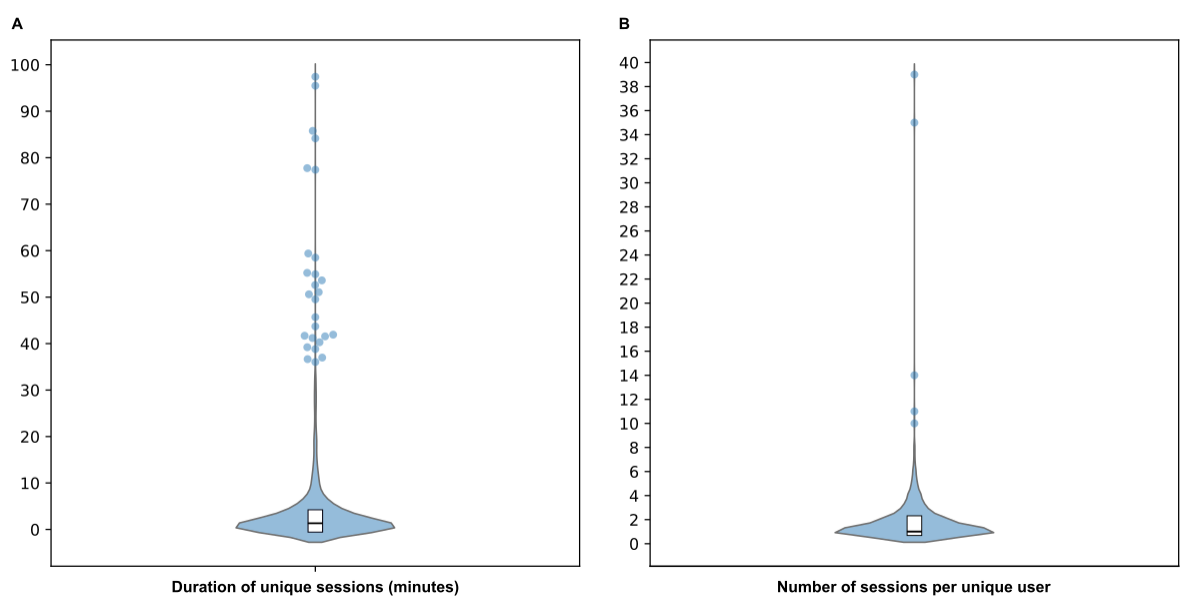
Analysis of user interaction with the Dolodoc mobile app based on the number of sessions and duration. Violin plots (box plots represent the IQR and the median) show (A) the distribution of the number of sessions per unique user and (B) the duration of each unique session. (A) 1473 sessions from Android users; (B) 3589 sessions from Android and iOS users. Outliers (above 95th percentile) are displayed as individual points. Underlying data are presented in Multimedia Appendix 4.

Over a 6-month period, from January to June 2024, a total of 3589 unique sessions were recorded, with 59% (2116/3589) on iOS and 41% (1473/3589) on Android. The mean duration of each session was 3 minutes and 21 seconds (201 s; Figure 3A and Multimedia Appendix 4 for percentile distribution). On average, 10.39 page views occurred per session. We observed 0.07% (25/37,255) missing values in the page titles data because these events were not counted as page views. Unique sessions containing only missing values were excluded from analysis (n=7) The bounce rate (ie, the percentage of sessions where only 1 page view occurred) was 23.34% (836/3582).

Among Android users, 1473 sessions were observed among 802 unique visitors. A total of 60.6% (486/802) of these visitors visited the app only once, and the mean number of sessions per visitor was 1.84, although a few outliers displayed more than 10 sessions during this period (Figure 3B and Multimedia Appendix 4 for percentile distribution). As mentioned in the methods section, iOS data were not available for this analysis.

Table 1 shows the top 10 most visited pages for all users. When navigating the app, the most visited page was the “first launch page,” showing that a substantial proportion of users used Dolodoc only once. Next, visitors most often viewed the home page and the page where they could monitor quality-of-life dimensions through a 4-point visual scale. Interestingly, the page where the strategies are compiled was the first action users took in the mobile app, because the 3 previous pages are automatically displayed upon connection. These results are consistent with the architecture and purpose of Dolodoc, guiding users toward self-observation and exploration of the included strategies for self-managing chronic pain.

**Table 1:** Top 10 most visited pages in Dolodoc over 6 months.

Page	Fraction of total views (n=37,230), n (%)	Functionality	Type of user interaction
First launch	9830 (26.4)	Welcome	Passive
Home	5737 (15.41)	Welcome and monitoring	Passive
Quality-of-life dimension evaluation	4333 (11.64)	Monitoring	Passive
Strategies	4312 (11.58)	Management	Active
Report export	2860 (7.68)	Monitoring	Active
Details of activities	2509 (6.74)	Management	Active
User profile	1479 (3.97)	Personalization	Active
Graphical view of collected data	843 (2.26)	Monitoring	Active
History of pain	789 (2.12)	Monitoring	Active
Dashboard	661 (1.78)	Monitoring	Active

Interestingly, the time spent on each page of the app does not follow the same architectural logic as the most visited pages (Table 2). Users spent the most time on less accessible pages (ie, pages that required more clicks to be accessed) of the app, such as a page where they could describe and add characteristics to their pain named “add your pain,” a page containing useful links, and a page where they could add their current medication named “add medication.” These results showed what users actively sought as they navigated the app, providing valuable feedback and insight into their expectations and needs.

**Table 2:** Top 10 most engaging pages in the Dolodoc mobile app based on average visit duration over 6 months.

Page	Time spent (s), mean (SD)	App content functionality	Type of user interaction
Add your pain	78.97 (107.23)	Personalization	Active
Useful links	59.70 (209.62)	Education	Active
Add medication	48.15 (62.67)	Personalization	Active
Association	39.65 (147.96)	Social support	Active
Home	39.08 (108.24)	Welcome and monitoring	Passive
Graphical view of collected data	28.89 (102.56)	Monitoring	Active
Create an activity	28.64 (69.30)	Management	Active
Report	23.71 (96.94)	Monitoring	Active
Print report	21.31 (59.46)	Monitoring	Active
Personal information	20.32 (27.91)	Monitoring	Active

## Discussion

### Key Results

This study provides a detailed evaluation of specific dimensions of UX, focusing on content acceptability and real-world use patterns, rather than on interaction design or interface usability. The population that evaluated the app’s content was unevenly affected by pain when assessing its impact on 7 quality-of-life dimensions, highlighting the variability in the experience of chronic pain and the need for personalized approaches to pain management. Self-management strategies included in Dolodoc were perceived as engaging by users, based on 5 acceptability criteria: understandability, motivational aspect, feasibility, relevance, and alignment with quality-of-life dimensions. This result was not straightforward, because although the app was developed using a user-centered design, end users were not involved in developing this content [23]. Although participants in the acceptability study perceived the content as acceptable and engaging, real-world use data showed that 60% (486/802) of users discontinued the app after a single visit. This suggests a potential discrepancy between perceived acceptability in an experimental context and actual acceptability in real-life conditions. However, users were actively engaged with less accessible features that required deeper navigation and/or active data entry, which may explain the longer visit durations. Personal pain and medication descriptions underscore the importance of being active with the app, and the need for personalization to enhance engagement and motivation [27]. Pages offering educational and social support were also among the most visited in the app, underscoring their relevance. Overall, this mixed methods pilot study provides preliminary insights into UX parameters of a mobile app for the self-management of chronic pain, highlighting directions for future research rather than serving as a comprehensive review of the topic.

### Limitations

The main limitation of the acceptability study is the relatively small number of patients (N=33), which limits generalizability; therefore, the results must be interpreted as preliminary. Additionally, patients were recruited primarily by health care professionals, which may lead to selection bias, with the most motivated patients participating. Further exploration of the difference between active and passive approaches would have brought valuable information to the study. However, our sample was diverse for demographics and pain-related characteristics.

A major limitation of this study is the lack of qualitative feedback from users who discontinued the app early. While the use analytics indicate that approximately 60% (486/802) of users accessed the app only once, the reasons underlying this early dropout remain unknown. Without direct user feedback, it is not possible to determine whether the discontinuation was due to usability issues, a lack of perceived relevance, insufficient onboarding, or unmet expectations. This limits our ability to fully interpret acceptability in real-world conditions, particularly for users who did not engage beyond an initial session. Taken together, these findings indicate that good content acceptability alone is not sufficient to ensure sustained use or even initial adoption, highlighting the multifaceted nature of UX in mHealth interventions.

Another limitation of the acceptability study is the assessment of tailored acceptability criteria, instead of a standard measurement instrument, such as the user version of the Mobile Application Rating Scale (uMARS) [33]. However, our study focuses on a French-speaking population, and at the time of the study’s conception, the uMARS had not been translated into French. As mentioned in the methods section, the main limitation of the use study was excluding iOS users from the number of sessions per visitor calculation. Furthermore, interpreting bounce rate and session data requires caution, as these metrics do not directly reflect user satisfaction or use.

### Interpretation

This study highlights the challenges of adopting a pain self-management app and identifies an important gap between acceptability and long-term engagement. This rare combination of real-world and experimental UX observations in the context of chronic pain provides useful, patient-centered insights that could help improve Dolodoc and inspire similar mHealth innovations. Indeed, postproduction UX studies in the context of mHealth solutions for chronic pain are sparse and usually based on small user samples, lacking real-world use data [24,34-36]. Using both ecological and survey-based data helps mitigate the “trial bias,” which has been previously described as an important confounder in user engagement studies of unguided eHealth interventions [34].

Users were satisfied with the content of this mHealth solution, suggesting that the user-centered design that guided its development [23] led to good acceptability of Dolodoc, similar to other mHealth interventions for chronic pain. In comparison, other therapeutic modalities, such as cognitive behavioral therapy [37], mindfulness-based interventions [38], physical activity [39], and multimodal group therapy [40], are also generally well accepted by patients experiencing chronic pain. However, real-world use habits in our study demonstrate the need to enhance long-term engagement further. This study does not identify specific barriers to adoption and sustained use; however, poor long-term engagement has already been described for other mHealth interventions in chronic disorders [16] and chronic pain [41], highlighting important barriers to use, such as the lack of reminders [26,42].

The diversity of pain profiles among our sample of users in the acceptability study, and the fact that global users actively spent most of their time on pages that were dedicated to personalization features, reveal the importance of personalizing pain-management mHealth solutions, in accordance with previous reports [25,26]. The personalization of mHealth apps positively influences users’ trust, thereby increasing the likelihood of their adoption [43].

### Generalizability

Despite the limitations mentioned, the study provides a strong initial assessment of the acceptability and use of Dolodoc. Although these UX results are specific to this mHealth solution, they align with reports on similar interventions regarding use [44,45] and acceptability [46].

### Perspectives

On the basis of the results of this study, it seems relevant to add features to improve engagement over time, such as a reward system and additional features [26]. Additionally, it would be useful to revise the app navigation logic to facilitate access to the most frequently used pages. Future improvements could also help us collect UX data longitudinally by letting users rate content within the app using like or dislike buttons.

Future research should prioritize qualitative investigations to understand the reasons for early discontinuation. Semistructured interviews or focus groups involving users who abandoned the app after initial use could provide valuable insights into barriers related to usability, perceived value, emotional experience, or contextual factors. Such qualitative feedback would complement use analytics and content acceptability measures, enabling a more comprehensive understanding of UX and informing more targeted design improvements.

However, optimizing digital therapeutic alliance [47] remains a key challenge for the future of mHealth. In this regard, digital navigation follow-ups have emerged as a potential solution, grounded in human interactions, to maximize patient engagement with mHealth solutions [48] and increase their clinical impact [49].

## Appendix

Multimedia Appendix 1 Example of screenshots of the Dolodoc mobile app from a previous study [30].

Multimedia Appendix 2 Example of a strategy and the evaluation items.

Multimedia Appendix 3 Mean ratings of adoption criteria (columns) by quality-of-life dimensions (rows).

Multimedia Appendix 4 Percentile distribution of user interaction.

## Abbreviations

mHealth: mobile health
uMARS: user version of the Mobile Application Rating Scale
UX: user experience
