# Supplementary material for: A Chronic Pain Self-Management Mobile App (Dolodoc): Cross-Sectional Acceptability Study
Source: JMIR Hum Factors. 2026 May 14;13:e77163. doi: 10.2196/77163 (PMC13219990; doi:10.2196/77163)

Appendix 1: Example of screens of the application Dolodoc, from a previous article ^30^

The left panel shows the welcome page, which depicts the seven quality-of-life dimensions as a tree that flourishes or wilts based on the user's ratings. The middle panel displays the strategies page. The right panel shows the coach-user interaction, where users rate a quality-of-life dimension.
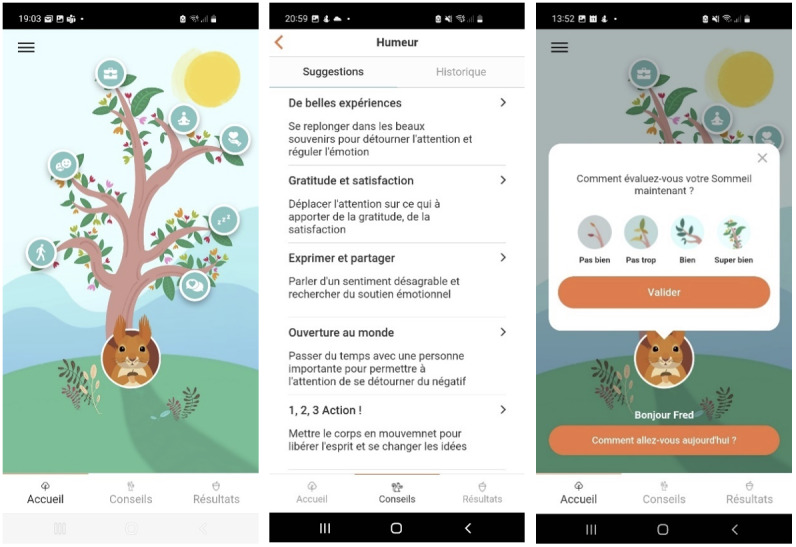

Supplement: Multimedia Appendix 1 [file humanfactors_v13i1e77163_app1.docx]
