# Supplementary material for: A Chronic Pain Self-Management Mobile App (Dolodoc): Cross-Sectional Acceptability Study
Source: JMIR Hum Factors. 2026 May 14;13:e77163. doi: 10.2196/77163 (PMC13219990; doi:10.2196/77163)
Supplement: Multimedia Appendix 2 [file humanfactors_v13i1e77163_app2.docx]

Appendix 2: Example of a strategy and the evaluation items.

| Communicate how you really feel to your spouse or partner and your loved ones. Daring to communicate what you are going through, without pretending otherwise, allows the other person to understand and support you better. It is advisable to express an emotion by speaking in the first person, explaining what triggers it and proposing a solution or at least discussing it. For example, you can use phrases such as (‘In this situation, I feel ... because ... and I need ...’).  Why?  A lack of communication can damage the quality of relationships. Giving incorrect information about your condition can lead the other person to doubt the seriousness and legitimacy of the situation, thereby increasing feelings of loneliness. Expressing your emotions not only improves emotional bonds and mutual understanding, but also helps to build intimacy between people and maintain a quality relationship. In addition, communication helps to find solutions and strengthens resources for overcoming various obstacles. | | | | | |
| --- | --- | --- | --- | --- | --- |
| How do you feel about this strategy, based on : | Strongly disagree | Disagree | Neutral | Agree | Strongly agree |
| Understandability |  |  |  |  |  |
| Motivational impact |  |  |  |  |  |
| Feasibility |  |  |  |  |  |
| Relevance |  |  |  |  |  |
| Relevant to the relationship dimension |  |  |  |  |  |
