# Supplementary material for: A Chronic Pain Self-Management Mobile App (Dolodoc): Cross-Sectional Acceptability Study
Source: JMIR Hum Factors. 2026 May 14;13:e77163. doi: 10.2196/77163 (PMC13219990; doi:10.2196/77163)
Supplement: Multimedia Appendix 3 [file humanfactors_v13i1e77163_app3.docx]

Supplementary table 1. Mean ratings of adoption criteria (columns) by quality-of-life dimensions (rows).

|  | Understandability | Motivational impact | Feasibility | Relevance | Alignement to the dimensions | *Mean* |
| --- | --- | --- | --- | --- | --- | --- |
| Mood | 1.46 | 1.09 | 1.26 | 1.11 | 1.27 | *1.24* |
| Social support | 1.48 | 1.18 | 0.91 | 1.08 | 1.28 | *1.19* |
| Daily activities | 1.56 | 1.20 | 1.21 | 0.99 | 1.40 | *1.27* |
| Sleep | 1.62 | 1.25 | 1.12 | 1.17 | 1.54 | *1.34* |
| Relaxation | 1.34 | 1.04 | 0.78 | 0.83 | 1.21 | *1.04* |
| Intimacy | 1.52 | 0.89 | 1.04 | 0.93 | 1.44 | *1.16* |
| Work | 1.41 | 1.02 | 0.68 | 0.79 | 1.30 | *1.04* |
| Mean | *1.48* | *1.09* | *1.00* | *0.99* | *1.35* |  |
